# Neuronal versus Glial CB2 Receptors: Insights from a Novel CB2-KO-eGFP Reporter Mouse Line

**DOI:** 10.21203/rs.3.rs-8297538/v1

**Published:** 2025-12-19

**Authors:** Zheng-Xiong Xi, Emily Linz, Hai-Ying Zhang, Christopher Dunn, Guo-Hua Bi, Ewa Galaj, Maia Maras, Bruce Hope, Francisco Javier Rubio, Qing-Rong Liu

**Affiliations:** National Institute on Drug Abuse; National Institute on Drug Abuse; National Institute on Drug Abuse; National Institute on Drug Abuse; National Institute on Drug Abuse; National Institute on Drug Abuse; National Institute on Drug Abuse; NIDA / NIH; National Institute on Drug Abuse; National Institute on Aging, National Institutes of Health

**Keywords:** CB2 receptor, neurons, astrocytes, microglia, CB2-KO-eGFP, CB2-KO, GFP reporter

## Abstract

The cannabinoid CB2 receptor (CB2R) has emerged as a promising therapeutic target for pain and central nervous system disorders, yet its brain expression has remained controversial due to low basal levels and the lack of reliable antibodies. Previous green fluorescent protein (GFP) reporter mouse lines have produced conflicting findings, possibly because GFP was either randomly inserted into the genome or placed in the 3'-untranslated region of the CB2R gene (*Cnr2*), complicating interpretation. Here, we report a new CB2-KO-eGFP mouse line in which the endogenous *Cnr2* coding region was precisely replaced with enhanced GFP through targeted knock-in, generating a combined CB2R knockout and GFP reporter. Loss of CB2R expression was confirmed by qRT-PCR, RNAscope in situ hybridization, and cannabinoid pharmacological assays. GFP-immunostaining was detected across multiple brain regions, including cingulate cortex, hippocampus, red nucleus, and cerebellum, and in several cell types such as microglia, astrocytes, and neurons. Flow cytometry revealed strong GFP signals in spleen and blood cells and quantifiable GFP expression in brain tissue. Notably, ~ 70% of microglia and ~ 4% of neurons in cortex and hippocampus expressed GFP under normal physiological conditions. These findings demonstrate that CB2R is indeed expressed in healthy brain tissue and across multiple neural and glial cell types, resolving long-standing uncertainty regarding CB2R localization. Functionally, CB2R deletion reduced cannabinoid-induced analgesia, hypothermia, and catalepsy, confirming the receptor’s physiological relevance. This new mouse line provides a reliable and highly informative tool for defining CB2R expression and function in both the brain and peripheral immune system.

## Introduction

The CB2 receptor (CB2R), encoded by *CNR2*, is a G protein-coupled receptor predominantly found in immune cells, where its activation regulates cytokine release and suppresses inflammation^1, 2^. CB2R was once considered a peripheral receptor^3^. However, recent studies also identified low level CB2R expression in the brain, where it modulates neuronal activity, neurotransmission, and neuroinflammation^1, 4, 5^. The minimal psychoactive effects of CB2R ligands in healthy subjects and the inducible expression of CB2R under pathological conditions make CB2R a unique therapeutic target with few adverse effects^6-8^. Extensive research highlights its role in neuroinflammation, and in neurodegenerative and neuropsychiatric disorders, positioning CB2R as a promising target for treating CNS diseases such as chronic pain, Alzheimer’s and Parkinson’s diseases, multiple sclerosis, schizophrenia, depression, and substance use disorders^1, 9-14^.

Despite significant progress in identifying CB2R function, its presence and cellular distribution in the brain under physiological and pathological conditions have been on debate for over three decades^1, 4, 7, 15-17^. This uncertainty is largely due to the low basal levels of CB2R expression in healthy brains^18-20^ and the poor specificity of many commercially available anti-CB2 antibodies^17, 21, 22^. When CB2R was first cloned in 1993, it was found primarily in peripheral immune cells^3^. Early follow-up studies supported this finding, failing to detect *Cnr2* expression in the healthy brain although CB2R was detected in the brain under pathological conditions such as Alzheimer’s and Parkinson’s diseases, and multiple sclerosis^15, 23, 24^. This view was challenged in 2005 by the discovery of *Cnr2* mRNA and protein in the brainstem neurons of rats, mice and ferrets under non-pathological conditions^25^. Since then, advances in genetic and molecular techniques have pointed to CB2R’s presence in various brain cell types – including microglia, neurons and astrocytes – and in diverse brain regions such as the striatum, cerebral cortex, hippocampus, midbrain, and amygdala ^1, 4, 5, 18, 19, 26-28^.

To further investigate the functional role of CB2R in the brain, several genetic approaches have been used, but two CB2R-driven GFP reporter mouse strains have yielded conflicting results. In the CB2-GFPTg line^29^, GFP expression was detected in microglia not in neurons, whereas in the CB2^EGFP/f/f^ line^16^, no GFP signal was observed in the healthy brain. Notably, GFP immunoreactivity in the latter strain was observed in hippocampal microglia of 3-month-old 5×FAD mice, a model of Alzheimer’s disease. The lack of detectable GFP expression in neurons and microglia under normal conditions has fueled long-standing skepticism about whether functional CB2R is truly present in the brain. Consequently, the identity and expression pattern of brain CB2R has remained contentious for more than 30 years, highlighting the need to understand the reasons behind these contradictory findings.

To help resolve these discrepancies, we examined the GFP knock-in strategies used in the two reporter strains. In the CB2-GFPTg line^29^, a GFP reporter cassette replaces the *Cnr2*-coding sequence within a bacterial artificial chromosome (BAC) that includes the CB2R promoter and regulatory elements. This BAC construct is then microinjected into embryos and randomly integrated into the genome, enabling GFP expression to be driven by the CB2R regulatory sequences within the transgene (Suppl. Figure 1A). Although GFP was detected in microglia, the endogenous *Cnr2* gene remained intact, raising the possibility that transgene-driven GFP expression may not accurately reflect endogenous CB2R expression.

In the CB2^EGFP/f/f^ line^16^, an internal ribosomal entry site (IRES) followed by the EGFP gene (IRES-EGFP) was inserted downstream of the *Cnr2* stop codon within the 3' untranslated region (3'-UTR), flanked by two loxP sites (Suppl. Figure 1B). This dual CB2-Cre and GFP reporter mouse line can be used for both conditional CB2R deletion and reporter studies. A major limitation of the IRES-EGFP strategy is that, although GFP is transcribed under the control of the endogenous CB2R promoter, its translation is driven by a viral-derived IRES. This configuration decouples GFP translation from endogenous CB2R regulation due to the presence of a stop codon between the CB2R coding region and the inserted IRES-EGFP cassette (Suppl. Figure 1B). Furthermore, insertion of loxP sites within the *Cnr2* gene may affect transcription. Therefore, GFP expression in this line may not accurately represent endogenous CB2R protein expression.

To overcome these limitations, we generated a new CB2-KO-eGFP mouse line (Ingenious Targeting Laboratory, https://www.genetargeting.com/). In this line, the entire *Cnr2* coding sequence (open reading frame, ORF) was replaced with enhanced GFP (eGFP) via homologous recombination, while preserving all native regulatory elements (Suppl. Figure 1C). This design produces a dual CB2R-knockout and GFP reporter line in which GFP expression is driven by the endogenous *Cnr2* promoter and translational control sequences, which should faithfully reflect native expression patterns.

To characterize this line, we used quantitative RT-PCR, RNAscope *in situ* hybridization, and cannabinoid pharmacology assays to confirm *Cnr2* deletion in both spleen and brain tissues. Immunohistochemistry (IHC) was then performed to assess GFP expression across brain regions and cell types, while flow cytometry quantified GFP-positive cells in both peripheral and brain tissues. We observed complete loss of CB2R expression and function, strong GFP signals in peripheral immune cells, and weaker but distinct expression in the cortex and hippocampus. Approximately 70% of microglia and ~ 4% of neurons and astrocytes were GFP-positive, compared with wild-type control mice. Behaviorally, CB2-KO-eGFP mice displayed increased basal locomotor activity and greater age-related weight gain as well as reduced behavioral and functional responses to D^9^-THC.

These new findings in the CB2-KO-eGFP mouse line help resolve a long-standing, three-decade debate over whether functional CB2R is expressed in the healthy, non-pathological brain and whether it is present in both glial cells and neurons. This genetically precise reporter line provides a reliable and physiologically relevant model for investigating CB2R expression and function across the central nervous system as well as in peripheral tissues.

## Results

### Generation of CB2-KO-eGFP Mice

**Figure 1:**
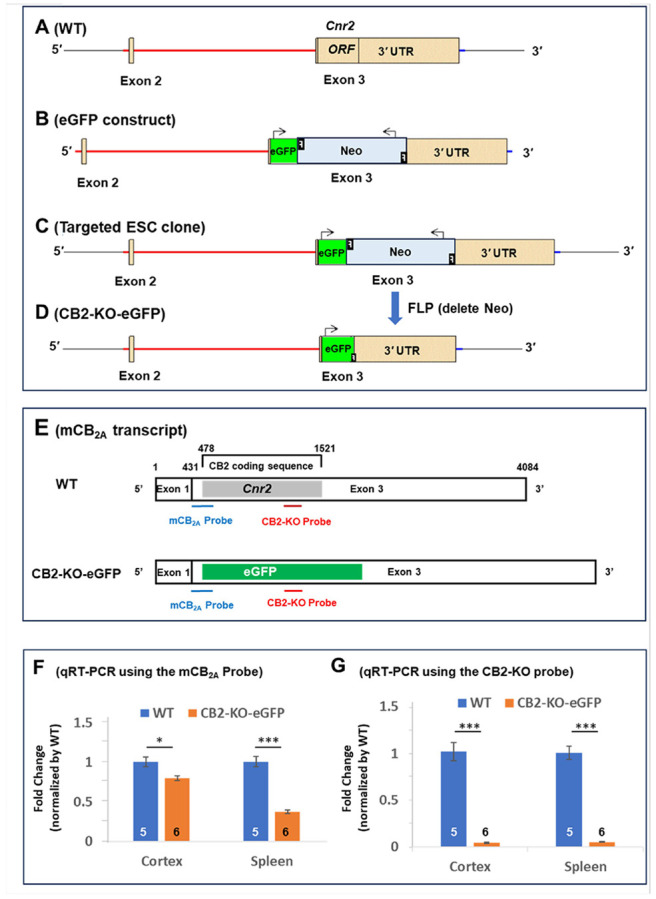
The GFP knock-in strategy in CB2-KO-eGFP mice and validation of CB2R knockout by quantitative real-time RT-PCR (qRT-PCR). (**A**) Schematic of the WT mouse *Cnr2* gene with three exons (Exon 1 not shown) with the *Cnr2* open reading frame (ORF) located in Exon 3. (**B**) eGFP-Neo construct used to replace the *Cnr2* ORF, with the Neo cassette flanked by two flippase (FLP) recognition target (FRT) sites (F = FRT sequence for FLP-mediated recombination in deleter ES cells). (**C**) Targeted embryonic stem cell (ESC) clone in which the eGFP-Neo cassette replaces the *Cnr2* ORF. (**D**) Modified *Cnr2* locus in CB2-KO-eGFP mice after homologous recombination and FLP-mediated deletion of the Neo cassette. A 79 bp “Neo footprint” (including one FRT site) remains and is targeted by a TaqMan probe for genotyping. (**E**) Schematic of two qRT-PCR probes targeting different *Cnr2* regions. (**F**) qRT-PCR using the mCB_2A_ probe shows markedly reduced *Cnr2* transcript levels in spleen and cortex of CB2-KO-eGFP mice. (**G**) qRT-PCR using the CB2-KO probe confirms complete loss of *Cnr2* mRNA in spleen and cortex of homozygous CB2-KO-eGFP mice relative to WT controls. (See also Suppl. Figs. 1, 2, 3; SI – the eGFP-KI strategy)

We generated the CB2-KO-eGFP mouse line through a contract with Ingenious Targeting Laboratory (genetargeting.com). To create this line, a genetically engineered mouse embryonic stem cell (ESC) line was used (Fig. 1). A custom targeting vector (eGFP construct) (Fig. 1B) was designed to replace the coding sequence (e.g. open reading frame, ORF) of exon 3 with the intact endogenous translation initiation site and stop codons of the mouse *Cnr2* gene encoding for CB2R (Fig. 1A). This knock-in eGFP was followed by a flippase recognition target (FRT)-flanked Neo selection cassette and the endogenous 3' untranslated region (UTR) (Fig. 1C). The targeting vector included a long homology arm (~ 5 kb) and a short homology arm (~ 2.6 kb) (Suppl. Figure 2) and was constructed by subcloning from a positively identified C57BL/6 fosmid clone using homologous recombination techniques (see the *Supplementary Information* (*SI*) – The eGFP-KI Strategy). Each modification step was validated by restriction analysis and sequencing.

The targeting vector was linearized and electroporated into a FLP 129 × C57BL/6 hybrid ESC line. After G418 antibiotic selection, resistant colonies were expanded and screened via PCR and sequencing to identify homologous recombinants. The Neo cassette was excised using flippase (FLP) recombinase during ESC expansion (Fig. 1D). Successfully targeted ESC clones (Fig. 1C) were microinjected into CD1 blastocysts and implanted into foster mothers. Chimeric offspring with high agouti coat color were bred with C57BL/6N WT mice. Tail biopsies were genotyped to confirm germline transmission of the targeted allele. Heterozygous CB2-KO-eGFP mice were then transferred to the NIDA IRP animal facility, where they were bred with C57BL/6N mice for up to 10 generations. Homozygous CB2-KO-eGFP mice were subsequently used for experiments. Heterozygous CB2-KO-eGFP mice were occasionally used for comparison of GFP expression with homozygous mice in the immunostaining experiment. Detailed eGFP-KI procedures are provided in the *SI* – The eGFP-KI Strategy.

### Validation of Loss of CB2R-Coding Sequence in CB2-KO-eGFP Mice

To validate *Cnr2* gene deletion, we performed qRT-PCR using two distinct TaqMan probes targeting different regions of the gene (Fig. 1E). Both probes detected *Cnr2* mRNA in the spleen and cortex of WT mice (Fig. 1F, G) (Suppl. Table 1). In CB2-KO-eGFP mice, results varied by probes. The mCB_2A_ probe, which spans the junction between the remaining *Cnr2* and GFP (Fig. 1E), showed significantly reduced CB2R mRNA levels compared to WT littermates (Fig. 1F). In contrast, the CB2-KO probe – specific to the deleted region of Cnr2 – detected CB2R mRNA only in WT mice, with barely detectable signal in CB2-KO-eGFP mice (Fig. 1G). These findings confirm successful deletion of the CB2R in the new transgenic line.

### RNAscope ISH Shows Absence of CB2R mRNA in CB2-KO-eGFP Mice

We next used RNAscope *in situ* hybridization (ISH) to assess *Cnr2* mRNA expression at the cellular level in peripheral (spleen) tissue and brain [ventral tegmental area (VTA) dopaminergic (DA) neurons] of CB2-KO-eGFP and WT mice. Midbrain DA neurons were selected due to their reliable labeling with tyrosine hydroxylase (TH), a specific neuronal marker.

Suppl. Figure 3A shows the mCB_2A_ transcript (isoform) structure and the RNAscope probe targeting the deleted exon 3 region of *Cnr2*. Using this probe, we observed *Cnr2* mRNA expression in the splenocytes of WT mice (Suppl. Figure 3B), but not in CB2-KO-eGFP mice (Suppl. Figure 3C). In the VTA, CB2R mRNA was detected at lower but consistent levels in TH^+^ DA neurons of WT mice (Suppl. Figure 3D), whereas no signal was observed in CB2-KO-eGFP mice (Suppl. Figure 3E). These results align with previous findings^18, 30^ and confirm the successful deletion of CB2R in this new CB2-KO-eGFP mouse strain.

### CB2-KO-eGFP Mice Exhibit Blunted Behavioral and Functional Responses to Δ^9^-THC

**Figure 2:**
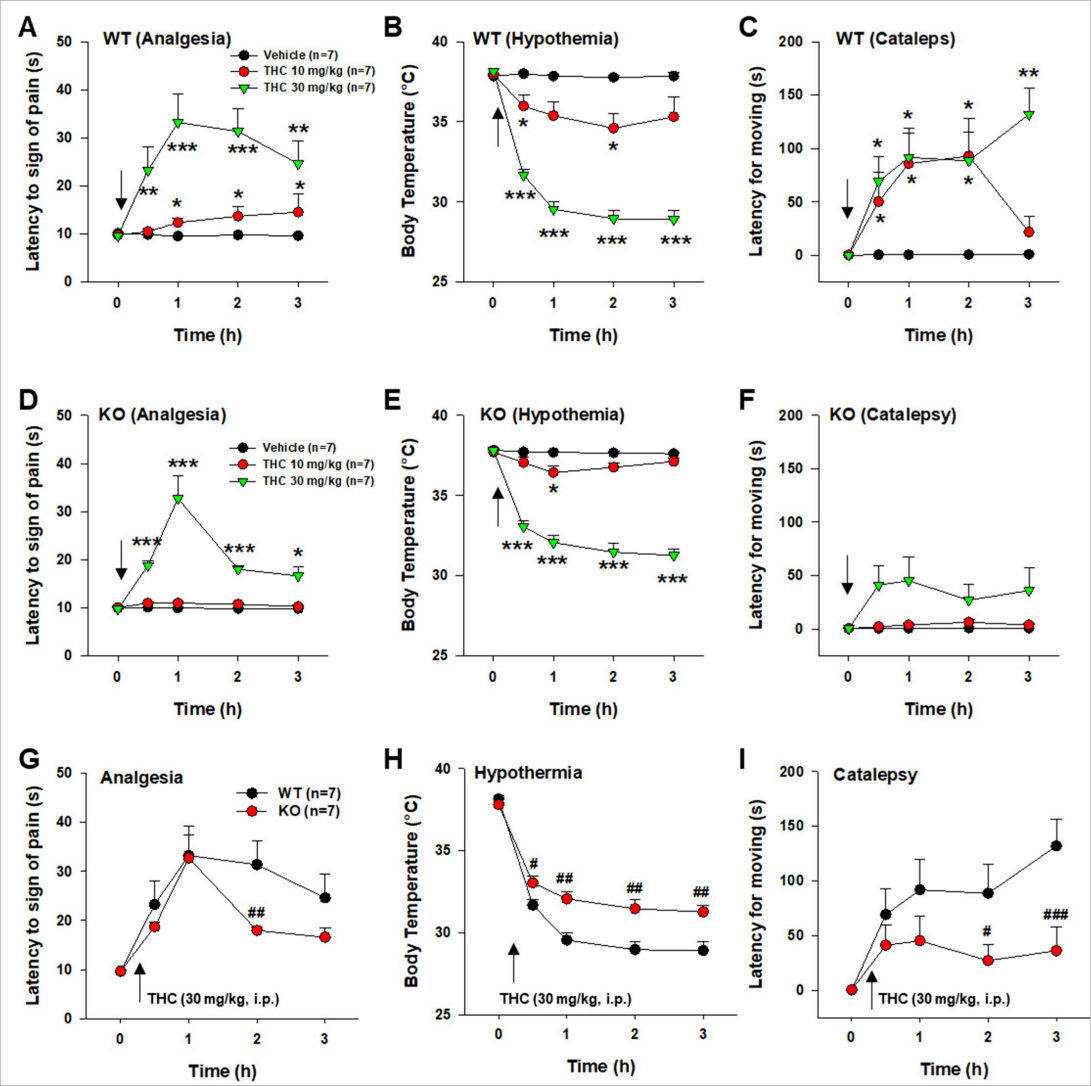
Behavioral and functional responses to Δ^9^-THC in WT and CB2-KO-eGFP mice. (**A–C**) Systemic Δ^9^-THC administration (10, 30 mg/kg, i.p.) produced dose-dependent effects on (**A**) hot-plate analgesia, (**B**) hypothermia, and (**C**) catalepsy in WT mice. (**D–F**) Systemic Δ^9^-THC also produced dose-dependent (**D**) analgesia, (**E**) hypothermia, and (**F**) catalepsy in CB2-KO-eGFP mice. (**G–I**) Comparison of the effects of 30 mg/kg Δ^9^-THC on (**G**) analgesia, (**H**) hypothermia, and (**I**) catalepsy between genotypes, showing significantly reduced responses in CB2-KO-eGFP mice compared to WT controls. *p<0.05, **p<0.01, ***p<0.001 compared to baseline before Δ^9^-THC injection; ^#^*p*<0.05, ^##^*p*<0.01; ^###^*p*<0.001 compared to WT group. (See Suppl. Fig. 4)

To assess the functional loss of CB2R in this mouse line, we evaluated the classic Δ^9^-THC-induced triad effects: analgesia, hypothermia, and catalepsy (Fig. 2). Systemic administration of Δ^9^-THC produced significant, dose-dependent effects in each measurement in both WT (Fig. 2A-C) and CB2-KO-eGFP mice (Fig. 2D-F). Two-way repeated measures (RM) ANOVA revealed significant Δ^9^-THC treatment main effect, time main effect, and treatment × time interactions in both genotypes (see Suppl. Table 2 for detailed F and p values). Post hoc pairwise analyses confirmed significant Δ^9^-THC effects in both groups (*p < 0.05, **p < 0.01, ***p < 0.001, compared to baseline, Fig. 2A-F).

However, compared to WT mice, CB2-KO-eGFP mice exhibited significantly attenuated responses to Δ^9^-THC across all three measurements (Fig. 2G-I). Two-way RM ANOVA revealed significant main effects of genotype, time, and genotype × time interactions (see Suppl. Table 2 for detailed F and p values). Post hoc analyses confirmed genotype differences in response magnitude (^#^*p* < 0.05, ^##^*p* < 0.01, ^###^*p* < 0.001, compared to WT, Fig. 2G-I).

We also compared open-field locomotor responses to Δ^9^-THC. Both groups of mice exhibited comparable Δ^9^-THC-induced hypoactivity, with no significant differences between genotype (Suppl. Figure 4), suggesting that deletion of CB2R does not significantly alter cannabinoid effects on open-field locomotor activity.

### GFP Expression Across Multiple Brain Regions

To assess GFP expression in the brain, we performed IHC using an anti-GFP antibody conjugated to Alexa^®^ Fluor 647 (AF-647). Supplementary Fig. 5 shows GFP immunostaining across whole coronal brain sections from WT and CB2-KO-eGFP mice, illustrating the regional distribution of GFP signal in the latter. Low-density, but reliably detectable GFP signals were observed in several brain regions of CB2-KO-eGFP mice, but not in WT mice, including the cingulate cortex, hippocampus (Hipp), midbrain red nucleus (RN) and mammillary body (MB) (Suppl. Figure 5).

**Figure 3:**
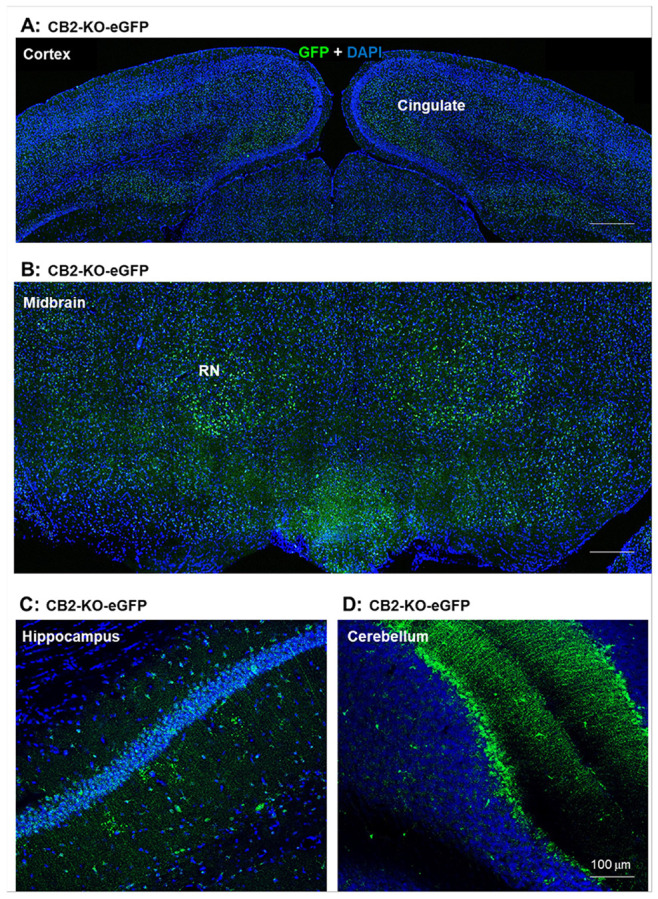
CB2R-driven GFP expression in the brain of homozygous CB2-KO-eGFP mice. (**A**, **B**, **C**, **D**) Representative confocal images of GFP-immunostaining in the cingulate cortex (layer 5) (**A**), midbrain red nucleus (**B**), hippocampus (**C**), and cerebellum (**D**), respectively, with high signal intensity in cerebellum and midbrain red nucleus. (Also see Suppl. Figs. 5-8)

**Figure 4:**
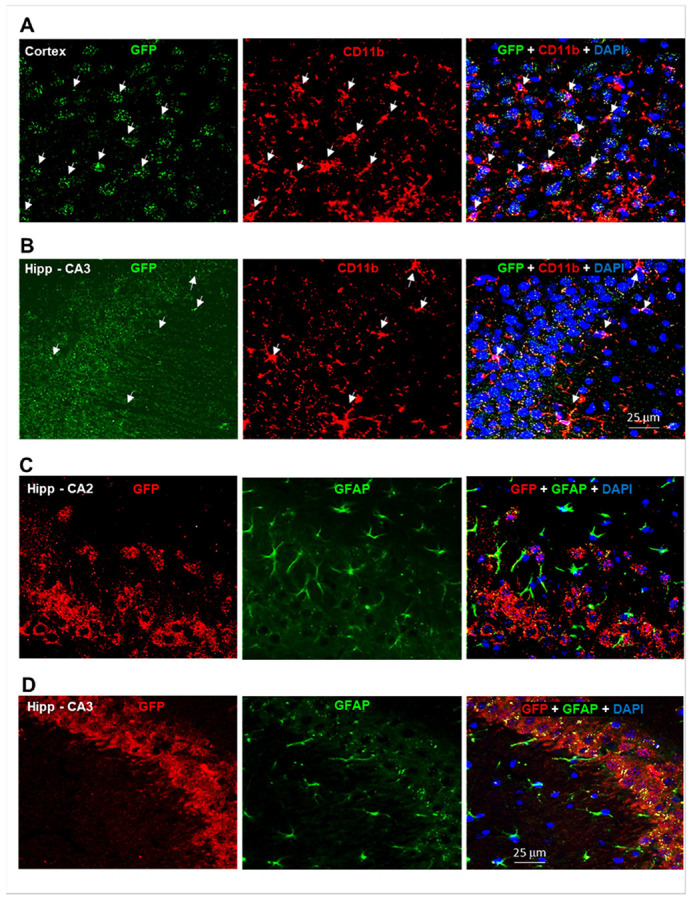
Cellular localization of GFP in glial cells and neurons. (**A**, **B**) Representative confocal images showing co-localization of GFP with CD11b in microglia in cortex (**A**) and hippocampus (**B**). (**C**, **D**) Representative images showing GFP co-localization with GFAP in astrocytes in hippocampal CA2 (**C**) and CA3 (**D**) subregions. Strong GFP signals are also detected in hippocampal neurons (**C**, **D**), although neuronal markers are not shown. (See Suppl. Figs. 9, 10)

Figure 3 presents high-magnification (10×) images from the same mouse, highlighting methodin the cingulate cortex (Fig. 3A), RN (Fig. 4B), hippocampus (Fig. 3C), and cerebellum (Fig. 3D) of homozygous (Hom) CB2-KO-eGFP mice. Clear qualitative differences in GFP expression were observed when comparing CB2-KO-eGFP mice with wild-type controls, particularly in the cingulate cortex and red nucleus (RN) (Supplementary Fig. 6) as well as in the VTA and NAc (Supplementary Fig. 7).

We also compared GFP signal intensity in the hippocampus and cerebellum between heterozygous (Het) and homozygous (Hom) CB2-KO-eGFP mice. GFP expression in the hippocampus was comparable between Het and Hom mice (Suppl. Figure 8A, B), whereas Hom mice showed stronger GFP signals in Purkinje cell somata and the granular layer of the cerebellar cortex (Suppl. Figure 8C, D). These results indicate that CB2R is actively or tonically expressed under physiological conditions and suggest that both Het and Hom reporter mice are suitable for studying CB2R expression in the brain.

### GFP Expression in Both Neurons and Glial Cells in CB2-KO-eGFP Mice

Previous studies using IHC and RNAscope ISH have reported CB2R expression in neurons^18, 30-35^; however, visualizing CB2R signals in glial cells remains technically challenging due to their small size (e.g. microglia), low expression levels under physiological conditions^36^ and the lack of specific CB2R antibodies^17, 22^. In contrast, GFP reporter mice circumvent the need for CB2R antibodies, allowing direct visualization of promoter-driven GFP. This approach enables detection of glial CB2R expression, including low or diffuse signals that may fall below the threshold of conventional methods.

We validated the expression of GFP in glial cells and neurons (Fig. 4). Immunostaining for microglia using CD11b antibody revealed colocalization with GFP in CD11b^+^ microglia in the cortex (Fig. 4A) and hippocampus (Fig. 4B) (Suppl. Figure 9). Immunostaining for astrocytes revealed GFP expression in glial fibrillary acidic protein (GFAP)^+^ astrocytes in the hippocampus of normal brains although at lower levels (Fig. 4C, D; Suppl. Figure 10).

In contrast, stronger GFP signals were observed in hippocampal neurons, particularly in the CA2 and CA3 cellular layers (Fig. 4C, D; Suppl. Figure 10). These observations in CB2-eGFP reporter mice are consistent with those using other approaches indicating CB2R gene and protein expression in midbrain dopaminergic neurons neurons^18, 30, 37^, RN glutamatergic neurons^31^, and NAc GABAergic neurons ^32^ in healthy adult mice. Together, these results indicate that both glial cells and neurons express CB2R under physiological conditions.

### Flow Cytometry Reveals GFP Cells in Peripheral and Brain Tissues

Because GFP^+^ neurons and glial cells are often co-localized within the same brain regions and have complex morphologies, quantitative analysis by IHC is technically challenging. To overcome this, we used flow cytometry to assess GFP^+^ cells in peripheral and brain tissues and to quantify the proportions of dissociated neurons, microglia, and astrocytes expressing GFP using cell type–specific markers

**Figure 5:**
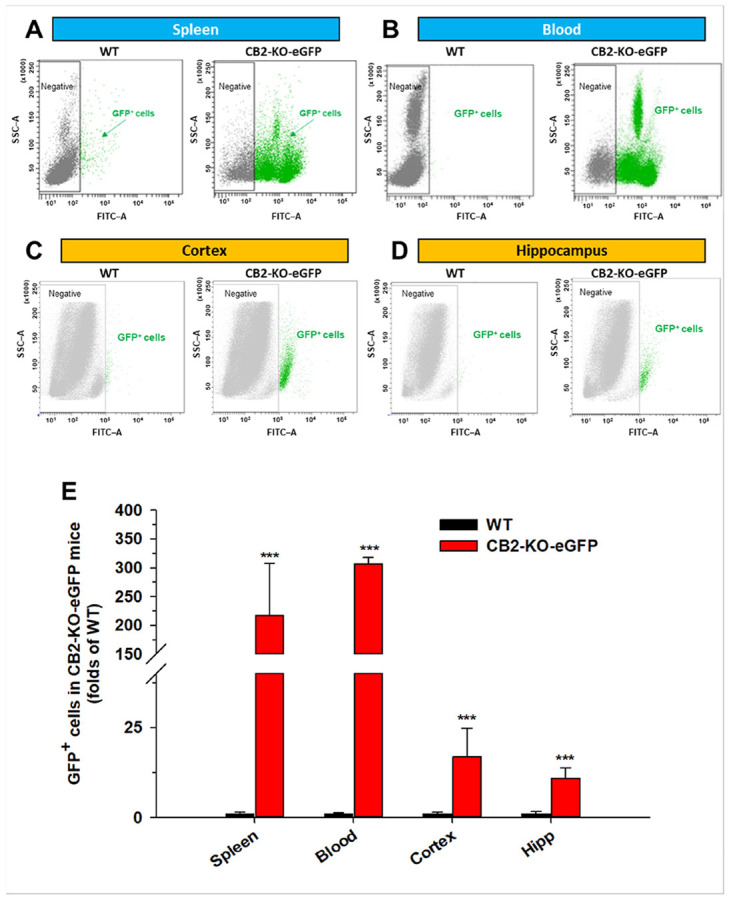
Flow cytometry analysis illustrates GFP expression in peripheral and brain tissues of CB2-KO-eGFP mice. Representative GFP fluorescence (FITC channel) vs. Side Scatter–Area (**SSC–A**) plots show distinct GFP^+^ cell populations (green) in each tissue compared with WT controls (gray, negative). Robust GFP expression was detected in splenocytes (**A**) and blood (**B**) cells, with lower but detectable levels in cortical (**C**) and hippocampal (**D**) cells in CB2-KO-eGFP mice. GFP-negative cells cluster near baseline fluorescence (left), whereas GFP-positive cells form a distinct right-shifted population (higher FITC). The GFP gate is set using WT controls to exclude autflouorescence. This prevents overestimation of GFP^+^ events from clumped cells. (**E**) The bar graph summarizes the relative abundance of GFP^+^ cells in CB2-KO-eGFP mice, expressed as fold change over WT levels. Data are presented as mean ± SEM (n = 2 per group). **FITC:** Fluorescein isothiocyanate, a fluorescent dye used to label gated GFP^+^ cells in singlets. (See Suppl. Fig. 11)

We first assessed the ability of flow cytometry to identify GFP^+^ cells in peripheral and brain tissues. Figure 5 shows GFP^+^ cells dissociated from the spleen, blood and brain tissues of WT and CB2-KO-eGFP mice. The results illustrate that a high density of GFP^+^ cells was observed in spleen and blood samples (Fig. 5A, B), while lower but clearly detectable GFP^+^ cells were also observed in brain regions such as the cingulate cortex (Fig. 5C) and hippocampus (Fig. 5D) in CB2-KO-eGFP mice. Such GFP^+^ cells were absent or barely detectable in WT controls. Quantitative analyses confirmed significantly higher numbers of GFP^+^ cells in both peripheral and brain tissues of CB2-KO-eGFP mice compared with WT mice (Fig. 5E).

Supplementary Fig. 11 presents the same results using alternative flow cytometry plots, showing a high density of GFP^+^ cells detected in the spleen and blood samples, and relatively lower levels of GFP^+^ cells in the cortex and hippocampus of CB2-KO-eGFP mice, wereas GFP^+^ cells were nearly undetectable in WT controls.

### Cellular Distribution of GFP Signal Using Cell Type-Specific Markers

To further characterize GFP-expressing cell types in CB2-KO-eGFP mice, we performed flow cytometry using fluorescent phycoerythrin (PE)-conjugated antibodies against NeuN (neurons), Ki67 (microglia), and GFAP (astrocytes), together with an Alexa^®^ Fluor 647–conjugated anti-GFP (AF647) antibody. PE^+^ and GFP^+^ cell populations were quantified from dissociated brain tissues.

**Figure 6:**
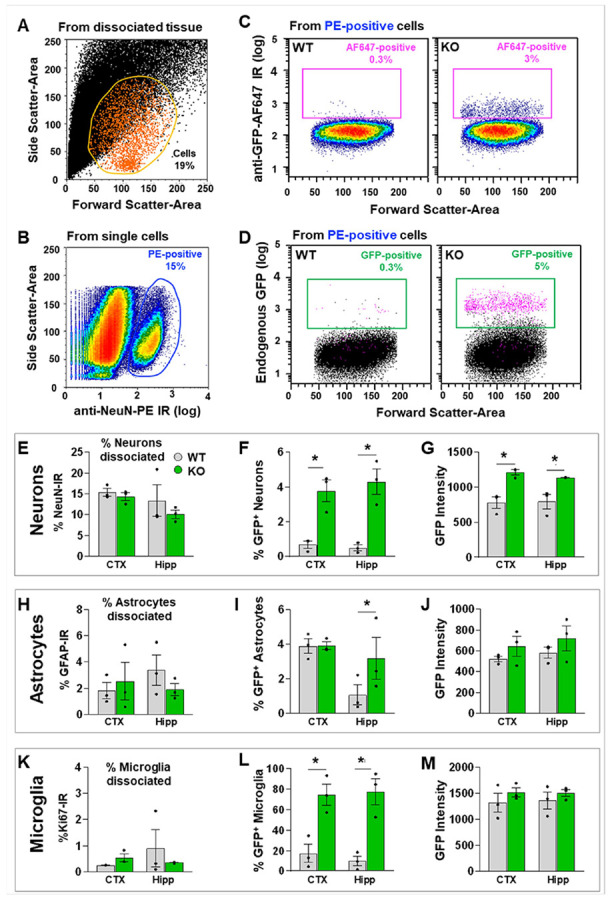
Flow cytometry analysis of GFP^+^ cells from the cortex and hippocampus of WT and CB2-KO-eGFP mice confirms GFP expression in both neurons and glial cells. (**A**) Representative linear plot of Forward Scatter – Area (X-axis, cell size) vs. Side Scatter – Area (Y-axis, granularity) showing that 19% of cells (orange gate) are positive for nuclear staining with DAPI (80–90% of nuclei were detected in all events; not shown). (**B**) Representative density plot of PE fluorescence (X-axis, logarithmic scale) following immunolabeling with PE-conjugated anti-NeuN antibody (neuronal marker), showing a blue gate containing 15% PE-positive events (neurons) from singlet cells (95% of all events; not shown). (**C**) Representative density plot of AF647 fluorescence (Y-axis, logarithmic scale) after immunolabeling with AF647-conjugated anti-GFP antibody, showing AF647-positive events (pink rectangular gate) from PE-positive neurons (as in **B**) in dissociated cortical tissue from WT or CB2-KO-eGFP (KO) mice. (**D**) Representative scatter plot of endogenous GFP fluorescence (Y-axis, logarithmic scale) from the same samples in C. Most GFP-positive events (pink dots, green gate) are also immunolabeled with anti-GFP-AF647 (pink gate in C). (**E–G**) Quantification from cortex (CTX) and hippocampus (Hipp) of WT (gray bars) and KO (green bars) mice: (**E**) % PE-NeuN–positive neurons, (**F**) % PE-positive neurons expressing GFP, and (**G**) GFP fluorescence intensity in neurons. (**H–J**) Quantification of astrocytes: (**H**) % PE–anti-GFAP–positive astrocytes, (**I**) % PE-positive astrocytes expressing GFP, and (**J**) GFP fluorescence intensity in astrocytes in CTX and Hipp. (**K–M**) Quantification of microglia: (**K**) % PE–anti-Ki67–positive microglia, (**L**) % PE-positive microglia expressing GFP, and (**M**) GFP fluorescence intensity in microglia in CTX and Hipp. IR, immunoreactivity; log, logarithmic scale. In density plots (**B**) and (**C**), red indicates the highest event density, and dark blue indicates the lowest. (See Suppl. Fig. 12)

Representative plots from single-cell suspensions are shown in Fig. 6A-D. We identified 19% DAPI^+^ cells (Fig. 6A) and 15% PE–NeuN^+^ neurons (Fig. 6B). Using AF647 anti-GFP, ~ 0.3% of NeuN^+^ neurons were GFP^+^ in WT mice *versus* ~ 3% in CB2-KO-eGFP mice (Fig. 6C). Without antibody amplification, endogenous GFP fluorescence showed similar results: ~0.3% GFP^+^ neurons in WT *versus* ~ 5% in CB2-KO-eGFP mice (Fig. 6D), confirming native GFP expression.

Quantitative analyses from three independent samples (two pooled mice per sample) are summarized in Fig. 6E-G. Among 10-15% NeuN^+^ neurons sorted (Fig. 6E), ~ 0.3% were GFP^+^ in WT cortex and hippocampus, compared with ~ 4% in CB2-KO-eGFP mice (Fig. 6F), a significant increase. GFP mean fluorescence intensity was also significantly higher in CB2-KO-eGFP mice (Fig. 6G).

Using the same approach (Suppl. Figure 12A–D), 2–3% of dissociated cells were GFAP^+^ astrocytes (Fig. 6H). In the hippocampus, ~ 3.5% of GFAP^+^ astrocytes were GFP^+^ in CB2-KO-eGFP mice *versus* < 1% in WT (Fig. 6I). No significant differences were detected in cortical astrocytes (Fig. 6I, J).

Flow cytometry also showed that 0.3–1% of cells were Ki67^+^ microglia (Fig. 6K; Suppl. Figure 12E–H). Among Ki67^+^ microglia, ~ 70% were GFP^+^ in the cortex and hippocampus of CB2-KO-eGFP, compared with 10–15% in WT mice (Fig. 6L). GFP mean fluorescence intensity did not differ significantly (Fig. 6M).

### CB2-KO-eGFP Mice Exhibit Baseline Hyperactivity and Increased Body Weight

Lastly, we compared behavioral phenotypes between CB2-KO-eGFP mice and their WT littermates. CB2-KO-eGFP mice showed signsignificantly increased baseline locomotor activity in the open-field test (Suppl. Figure 13A, B) and displayed greater body weight beginning around 6 months of age (Suppl. Figure 13C, D). Despite these weight differences, food intake and feeding behavior were comparable between genotypes (Suppl. Figure 13E). Nociceptive responses, assessed by the hot-plate test, also did not differ between CB2-KO-eGFP and WT mice (Suppl. Figure 13F).

## Discussion

In this study, we generated and validated a novel CB2-KO-eGFP mouse line in which the *Cnr2* open reading frame was replaced with an eGFP reporter. Molecular assays (qRT-PCR, RNAscope) and cellular analyses (IHC, flow cytometry) confirmed complete loss of CB2 receptor expression and robust GFP labeling in splenocytes, neurons, and microglia, with lower expression in astrocytes across multiple brain regions. Behaviorally, CB2-KO-eGFP mice showed increased baseline locomotion, elevated body weight, and markedly blunted behavioral and functional responses to Δ^9^-THC, supporting an essential role for CB2 receptors in basal physiology and cannabinoid signaling.

### CB2R in Neuropsychiatric Disorders

Over the past three decades, CB2R signaling has been implicated in multiple neuropsychiatric disorders^1, 7, 9, 13^. Clinical studies have linked elevated CB2R expression or *CNR2* polymorphisms to altered risk or symptom severity in schizophrenia^38^, depression^20^, Parkinson’s^39^ and Alzheimer’s diseases^40^. Postmortem analyses show CB2R upregulation or downregulation in microglia-rich brain regions, correlating with neuropathological severity^40, 41^. In animal models, CB2R activation reduces neuroinflammation, rescues synaptic plasticity, and alleviates stress-induced behavioral deficits^42, 43^. In schizophrenia-like states, CB2R agonists regulate dopaminergic and glutamatergic signaling, improving cognition and social function^27, 38, 44^. In substance use models, CB2R activation reduces self-administration and relapse-like behavior^35, 45, 46^, whereas CB2R deletion promotes drug-seeking^1, 47^. Neurodegenerative models show CB2R-mediated neuroprotection via suppression of microglial overactivation, enhancement of toxic aggregate clearance, and preservation of neuronal integrity^48, 49^. Given its relatively low brain expression compared to CB1R, CB2R is considered a promising therapeutic target with minimal psychoactive liability^15^. Numerous selective CB2R agonists have demonstrated therapeutic efficacy and favorable safety profiles in preclinical and clinical studies targeting chronic pain, neuroinflammation, and addiction^1, 11, 12, 14^. Nonetheless, the neural mechanisms underlying CB2R’s therapeutic effects remain incompletely understood.

### The “Identity Crisis” of Brain CB2R for Three Decades

CB2R was once thought to be absent from the brain^3^, but accumulating evidence now demonstrates its presence in microglia, astrocytes, and neurons under both physiological and pathological conditions^1, 4, 5, 15, 18, 25, 50^. In the healthy brain, CB2R expression is generally low but inducible during neuroinflammatory, pharmacological treatment, and neurodegenerative states^4, 7, 32, 49, 51, 52^. Functional studies suggest that CB2R modulates neuroimmune signaling, synaptic plasticity, and neurotransmitter release^1, 18, 27, 33, 53^, thereby influencing cognition, mood, and reward processing.

Despite these findings, the presence of CB2R in the brain remains controversial due to persistent methodological limitations. Many commercially available CB2R antibodies lack specificity, often producing signal even in CB2-KO tissue^15, 21, 22^. PET imaging with CB2R-selective ligands also shows minimal binding in healthy brains but robust increases during neuroinflammation. However, interpretation of these PET findings is complicated by radioligand off-target binding and species-dependent differences in ligand affinity^54, 55^. Moreover, two CB2-GFP reporter mouse lines have yielded inconsistent results, as we described above. These discrepancies have fueled debate over whether microglial CB2R expression is constitutive or only induced in reactive states, and whether CB2R is expressed in neurons and contributes to physiological and pathological conditions. Such uncertainties have hindered mechanistic understanding and translational progress.

### Discovery of Neuronal and Glial GFP Expression in This New Reporter Mice

The reasons underlying inconsistent findings from previous CB2R reporter lines remain unclear. Closer examination of their GFP knock-in strategies reveals inherent limitations, as noted above, suggesting that the GFP expression observed in those lines may not accurately report endogenous CB2R expression.

To address these issues, we generated a new CB2-KO-eGFP strain using homologous recombination in embryonic stem cells (ESG). The entire coding sequence of exon 3 of *Cnr2* was replaced with an eGFP-Neo cassette flanked by FRT sites, leaving endogenous promoter and untranslated regions intact. After FLP-mediated excision of the Neo cassette, targeted ESCs were used to generate germline-transmitting chimeras, which were subsequently backcrossed onto a C57BL/6N background for over 10 generations. This design preserves physiological promoter regulation of the reporter while creating a functional CB2R null allele.

Extensive molecular validation confirmed the deletion of *Cnr2* transcripts in homozygous mice and robust GFP expression in peripheral and brain tissues, including the cingulate cortex, hippocampus, red nucleus, and cerebellum. GFP signal was detected in microglia, astrocytes, and neurons. Flow cytometry analysis revealed that ~ 70% of Ki67^+^ microglia expressed GFP, along with smaller but significant proportions of neurons (~ 4%) and astrocytes (~ 4%), greatly exceeding baseline levels (< 1%) in WT controls. These findings support the existence of tonic CB2R expression across multiple cell types under physiological conditions.

We note that the proportion of GFP^+^ neurons (~ 4%) measured by flow cytometry is lower than that observed by IHC and RNAscope, both in this study and prior worlk^18, 22, 30, 33^. The reasons are unclear, but differential susceptibility to enzymatic and mechanical dissociation likely contribute^56, 57^. Neurons and astrocytes are more vulnerable to cell loss or quenching of GFP fluorescence during dissociation, whereas microglia remain robust and maintain strong GFP signal. Consequently, single-cell suspensions may not fully capture all cell populations or their intact fluorescence. Thus, the low percentages of GFP^+^ neurons or astrocytes may in part reflect technical underdetection.

Functionally, CB2-KO-eGFP mice exhibited baseline hyperactivity, increased body weight, and attenuated Δ^9^-THC-induced analgesia, hypothermia, and catalepsy. These findings align with our previous report demonstrating that deletion of CB2R in another CB2-KO strain similarly reduced cannabinoid-induced behavioral responses^58^. Notably, both genotypes showed comparable reductions in Δ^9^-THC–induced open-field locomotion. Together, these results validate the functional knockout and underscore the role of CB2R in modulating cannabinoid-induced analgesia, thermoregulation, and motor suppression, but not in basal locomotor activity.

### Advantages and disadvantages of the New CB2-KO-eGFP Mice

This targeted knock-in strategy offers several key advantages over existing CB2R reporter mice. Unlike BAC transgenic lines, it avoids random genomic insertion and dependence on non-native promoters. In contrast to IRES-based knock-ins, it eliminates translation-level decoupling by replacing the entire CB2R-coding sequence with a single eGFP reporter under the control of the endogenous *Cnr2* promoter. This design ensures faithful visualization of *Cnr2* transcriptional activity while simultaneously generating a complete functional knockout, enabling integrated anatomical, molecular, and behavioral analyses within the same animal.

Several limitations should be acknowledged. First, all experiments were performed in healthy mice; GFP expression dynamics under pathological conditions remains unexplored. Second, strong GFP signals were observed in the cerebellum and midbrain red nucleus relative to the cortex and hippocampus, but the proportion of GFP^+^ neurons in these GFP-rich regions was not quantified by flow cytometry. The percentage of GFP^+^ neurons or astrocytes may be higher than that in the cortex or hippocampus. Third, the embryonic deletion of *Cnr2* may induce compensatory changes during development that alter GFP expression or cellular phenotypes.

In summary, the CB2-KO-eGFP mouse offers a powerful tool for resolving the long-standing controversy surronding CB2R expression in the brain. By coupling endogenous promoter–driven GFP reporting with a genetic knockout, this model enables precise identification of CB2R-expressing cells while allowing direct functional assessment of CB2R loss. Its dual capacity for cell-type–specific localization and mechanistic interrogation should greatly advance understanding of CB2R biology in both peripheral and central systems. Ultimately, this strain is well positioned to accelerate discoveries in neuroimmune research, clarify CB2R contributions to CNS disorders, and guide CB2R-based therapeutic development.

## Materials and Methods

### Animals

Male and female CB2-knockout-eGFP reporter (CB2-KO-eGFP) mice, age of 8–24 weeks, generated by Ingenious Targeting Laboratory (Ronkonkoma, NY, USA) (https://www.genetargeting.com/) (see *SI* for the detail procedures), and their wildtype littermates were used in this study. Animals were housed in climate-controlled animal colony rooms on a 12-hr reversed light-dark cycle (lights on at 7:00 p.m., lights off at 7:00 a.m.) with free access to food and water throughout the study. The housing conditions and animal care were consistent with the Guide for the Care and Use of Laboratory Animals (National Research Council, 2011). All experimental procedures were approved by the National Institute on Drug Abuse Animal Care and Use Committee.

### Experiment 1: qRT-PCR

The quantitative real-time PCR (qRT-PCR) assay of brain CB2 mRNA levels was performed as described previously ^18, 20^. Because immune cells in blood contain a high density of CB2R, all mice used for qRT-PCR were perfused transcardially with 30–50 mL 0.9% saline under deep anesthesia, to prevent contamination of brain tissue by blood cells. Then brain and spleen were removed, and the prefrontal cortex and spleen were dissected. Two specific CB2R probes were used: mCB_2A_ TaqMan probe (Mm00438286_m1 that targets to the region 69–160 bp of X86405.1 (https://www.ncbi.nlm.nih.gov/nuccore/X86405.1) and a custom-designed CB2-KO TaqMan probe that recognizes the *Cnr2* gene-replaced region (1,877–2,820 bp of the Mus *Cnr2* mRNA sequence) in the exon 3 of *Cnr2* gene in CB2-KO-eGFP mice^18^. Mouse Gapdh mRNA detected by a commercially available Gapdh TaqMan probe (Mm99999915_g1) served as an endogenous control. The specific base pair sequences of the minor groove binder (MGB)-TaqMan probes and the primers used to detect CB2R mRNAs are listed in Suppl. Table S1.

### Experiment 2: RNAscope In Situ Hybridization (ISH)

RNAscope ISH was performed as previously described^18, 30^. RNAscope *in situ* hybridization of mouse brain and spleen sections was performed to further confirm CB2R gene loss in the present strain of CB2-KO-eGFP mice. Mice (WT and CB2-KO-eGFP; 3 mice aged 2–3 months of each genotype) were deeply anesthetized, and the whole brain was removed and rapidly frozen on dry ice. Fresh-frozen tissue sections (16–18 μm thick) were mounted on positively charged microscopic glass slides (Fisher Scientific) and stored at − 80°C until RNAscope ISH assays could be performed. A mouse *Cnr2*-specific RNA probe (RNAscope probe: Mm-*Cnr2*-O_2_, cat# 436091) that targets coding sequence (291–719 bp) of the Mus *Cnr2* mRNA sequence (NM_009924.3) and a TH-specific RNAscope probe (Cat #: 317621-C2, targeting 483–1,603 bp of the Mus musculus TH mRNA sequence, NM_009377.1) were designed and provided by Advanced Cell Diagnostics (Newark, CA, USA). The RNAscope mRNA-staining steps were performed following the manufacturer’s protocols. After a short incubation with DAPI (30 s), each slide received fluorescent mounting medium (Fluoro-Gel; #17 985, Electron Microscopy Science) and a coverslip. A Keyence BZ-X800 Fluorescence Microscope was used to take images at 60× magnification. Image Processing and Analysis by Java (ImageJ, NIH) software was used to quantify mRNA signals in the sections.

### Experiment 3: GFP-Immunohistochemistry

Mice were anesthetized with isflourane gas and intracardially perfused with ice-cold 0.9% saline then 4% PFA. Brains were removed and placed in 4% PFA overnight. The following morning, the tissue was transferred to a 20% sucrose solution in PB for 24 hours then a 30% sucrose solution in PB. After 48 hr in sucrose, brains were frozen and sliced in 30 μm coronal sections. Free floating slices were rinsed in PB (x5, 10 min each) and blocked in a 5% donkey serum and 0.3% Triton X-100 solution in PB for 1 hr with agitation. Sections were shielded from light and incubated overnight at 4°C on a shaker with two primary antibodies in PB containing 3% donkey serum and 0.3% Triton X-100. The following antibodies were selected: 1) Alexa Fluor^®^ 647 anti-GFP Antibody (1:200, BioLegend, Cat. # 338006), 2) Alexa Fluor^®^ 488 anti-Tyrosine Hydroxylase Antibody (1:1500, Biolegend, Cat. # 818005). Sections were rinsed in PB (x3, 10 min each) and mounted on gelatin-coated slides. Dapi-Fluoromount-G^™^ (Electron Microscopy Sciences, Cat. # 17984-24) was applied, and slides were cover slipped and allowed to dry in a dark place. Images were obtained using the Leica THUNDER microscope at 40x to create stitched images. The large volume computational clearing (LVCC) software on the THUNDER imager was also used.

To assess GFP colocalization with TH, CD11b (a microglial marker), or GFAP (an astrocytic marker), mouse brains were sectioned at 40 μm thickness using a Vibratome. Free-floating sections were washed three times in 1× PBST (1× PBS with 0.3% Triton X-100; 15 min each) and then incubated at room temperature for 2 h in blocking solution (10% normal donkey serum in 1× PBST). Sections were subsequently incubated overnight at 4°C with rabbit anti-GFP (Invitrogen, A-11122, 1:500) together with one of the following primary antibodies: mouse anti-TH (Millipore Sigma, T-1299, 1:1,000), mouse anti-CD11b (Cell Signaling, 46512S, 1:500), or mouse anti-GFAP (Invitrogen, A-21282, 1:1,000). The next day, sections were washed three times in 1× PBST (15 min each) and incubated for 2 h in the dark with Alexa Fluor 488- or 555-conjugated secondary antibodies. Finally, sections were mounted using Vectashield Vibrance^®^ Antifade Mounting Medium with DAPI (Vector Laboratories, Cat# H-1800). Confocal images were acquired on a Zeiss LSM 510 confocal microscope at the Light Imaging Facility, National Institute of Neurological Disorders and Stroke (NINDS).

### Experiment 4: Flow Cytometry

#### Peripheral Blood Cell Preparation

Wild-type (WT) and CB2-KO-eGFP mice were anesthetized with isflourane and euthanized by decapitation within 60 seconds. Approximately 100 μl of peripheral blood was collected into EDTA-coated tubes to prevent coagulation, gently inverted several times to ensure proper mixing, and either processed immediately or stored on ice. Blood samples were transferred into 10 ml of ACK (Ammonium-Chloride-Potassium) lysing buffer (Cat# A1049201, ThermoFisher) and incubated at room temperature for 30 minutes to lyse red blood cells. Following lysis, samples were washed by filling the tube with PBS and centrifuging at 500 × g for 5 minutes. The resulting cell pellet was resuspended in 20 ml PBS and centrifuged again to halt further lysis. After removing the supernatant, the pellet was gently resuspended in ~ 500 μl PBS and kept on ice until flow cytometry analysis.

#### Peripheral Tissue Preparation

To assess the presence of GFP^+^ cells in other CB2-rich peripheral immune tissues, mice were perfused with saline to remove circulating immune cells. Spleen was collected, finely minced on ice using razor blades, and transferred to 1 ml of ice-cold Hibernate A (HA-if; Brain Bits). After centrifugation at 110 × g for 2 minutes at 4°C, 1 ml of Accutase (SCR005; Millipore) was added, gently mixed by pipetting four times, and incubated for 30 min at 4°C with end-over-end rotation. Samples were then centrifuged at 960 × g for 2 minutes at 4°C, and the pellet was resuspended in 0.6 ml ice-cold Hibernate A. Cells were dissociated by sequential trituration using fire-polished glass pipettes with decreasing inner diameters (1.3 mm, 0.8 mm, and 0.4 mm), followed by three additional rounds using 0.4 mm pipettes. Each trituration step consisted of 10 gentle passes and a 2-minute rest on ice to allow debris to settle. Supernatants were pooled, yielding ~ 3.6 ml of dissociated cells.

After centrifugation (1,700 × g, 4 min, 4°C), the pellet was resuspended in 0.7 ml cold PBS and filtered sequentially through 100 μm and 40 μm cell strainers (BD Biosciences).

#### Brain Tissue Preparation

Brain dissection and cell preparation followed previously published protocols ^59-61^, with minor modifications. Mice were deeply anesthetized with isflourance, then perfused with cold PBS for 3 min to flush out blood cells. Immediately after brain extraction, 2-mm thick coronal sections containing cortical and hippocampal regions (Bregma − 4.4 mm to − 2.4 mm) were sliced. The cingulate cortex and CA1/CA2 hippocampus were dissected on ice and transferred into a 1.5 ml microtube containing 1 ml of ice-cold Hibernate A.

Tissues from two mice were pooled and manually triturated using a plastic pipette tip, followed by serial trituration with fire-polished glass pipettes of decreasing diameters (1.3 mm, 0.8 mm, and 0.4 mm). Each step involved 10 gentle passes to generate a cloudy suspension of dissociated cells. Samples were split into two tubes for fixation/permeabilization by adding an equal volume of 100% cold ethanol (− 20°C), incubated on ice for 7 minutes with inversion after 3 minutes. After centrifugation (1,700 × g, 4 minutes, 4°C), the pellet was resuspended in 0.7 ml cold PBS and filtered through 100 μm cell strainers.

#### Neuron and Glial Cell Immunostaining

To determine the proportion of GFP-expressing neurons and glial cells, immunostaining with fluorescent antibodies was performed. The cell suspension was divided into three 1.5 ml microtubes and incubated for 30 minutes at 4°C in 0.7 ml PBS with the following antibodies: All tubes, Alexa^®^ Fluor 647-conjugated anti-GFP antibody (1:250, BioLegend, #338006); Tube 1, PE-conjugated anti-NeuN antibody (1:500, Millipore, FCMAB317PE) for neurons; Tube 2, PE-conjugated anti-GFAP antibody (1:500, Cell Signaling, #12389) for astrocytes; Tube 3, PE-conjugated anti-Ki67 antibody (1:500, Cell Signaling, #12160) for microglia. After incubation, cells were washed twice with 0.8–1 ml cold PBS (1,300 × g, 3 minutes, 4°C), resuspended in 0.5 ml cold PBS, and filtered through 40 μm cell strainers for sorting.

#### Flow Cytometry

Cell sorting was performed using a FACSAria Fusion SORP flow cytometer (BD Biosciences). DAPI (1 μg/ml) was used to identify nucleated cells, with ~ 80–90% of events in the ‘Cell’ gate being DAPI-positive. Doublets were excluded using a restricted gate based on forward scatter width vs. height, with > 95% of events confirmed as single, DAPI-positive cells. Final gating and data analysis were conducted offline using FCS Express 7 (De Novo Software).

### Experiment 5: Open-field locomotion

To determine whether CB2R deletion alters locomotor response to Δ^9^-THC, we injected vehicle or Δ^9^-THC (0, 10, and 30 mg/kg) to CB2-KO-eGFP mice and their wildtype littermates and measured locomotion behavior in the open-field test. Animals were given two consecutive day sessions (1 hr) in the open-field chambers for habituation and minimization of novelty exploratory behavior. Then, on the following test days, animals were placed in the open-field chambers for 1 hr prior i.p. injections for baseline locomotion measurements. After baseline, animals were injected with one dose of Δ^9^-THC or vehicle and then immediately placed in the open-field apparatus to obtain locomotion measurements after injections for 2 hrs. The experiment was conducted in a within-subjects design with Δ^9^-THC doses counterbalanced and at least 2–3 days of time interval between test days.

### Experiment 6: Food pellet self-administration

To determine whether deletion of CB2R alters food taking and body weight, mice were trained on a daily 1 h FR1 schedule for 1–2 weeks until reliable response was achieved. A rodent diet food pellet (LabTab Ain-76A, TestDiet) served as a reinforcer. Each pellet is 45 mg and contains 5.1% fat, 65.2% carbohydrate, 4.8% fiber, and 2.9% ash contents. For each session, both response levers extended into the chamber. The light cue-paired “active” lever delivered 1 pellet per press and the “inactive” lever failed to elicit cues or reward delivery. Sessions were terminated upon reaching the pellet delivery maximum (60 pellets) or after 1 h had elapsed.

### Experiment 7: Δ^9^-THC-induced triad effects

The procedures for measurement of THC-induced triad effects were the same as we previously reported ^62^. Briefly, CB2-KO-eGFP and their wildtype littermates (n = 8 per group) were treated with vehicle or Δ^9^-THC (10 or 30 mg/kg, i.p.) to measure cannabinoid-induced analgesia, catalepsy, and hypothermia. Measurements were taken 0.5 hr before and 0.5, 1, 1.5, and 2 hrs post Δ^9^-THC injection on the testing day. The order of testing was counterbalanced. Time intervals between test days were two to three days.

#### Analgesia

Thermal nociception was measured using a hot plate device (Model 39, IITC Life Science Inc., CA). Mice were placed on a hot plate heated to 52°C with a transparent barrier in place. The latency to exhibit the first thermal nociceptive sign, including paw licking, stomping or shaking hind paws, and jumping, was recorded to the nearest hundredth of a second. Mice were removed from the hot plate immediately after the first thermal nociceptive sign or, if no thermal nociceptive signs occurred, at 60 seconds to avoid tissue damage.

#### Hypothermia

To measure changes in body temperature, the RET-2 rectal probe (Harvard Apparatus, Holliston, MA) was lubricated with seed oil and gently inserted 2 cm into the rectum. Temperature was recorded once the measurement stabilized, to the nearest tenth of a °C.

#### Catalepsy

Cataleptic behavior was measured using an elevated bar test. Subjects’ front paws were placed on a metal bar at a height where their hind paws just reached the ground. The latency for the mice to remove both front paws from the bar and place them on the ground was recorded to the nearest tenth of a second, with a cutoff of 120 s.

#### Drugs

D^9^-THC was obtained through the NIDA Pharmacy and was dissolved in 5% Cremophor.

### Data analysis

All data are represented as the mean ± SEM. Animal group sizes were chosen based on power analysis (n ≥ 8 per group) and extensive previous experience with the animal models used. The group size is the number of independent values (individual animal). To validate the use of parametric statistics, we ensured that the residuals were normally distributed (Shapiro Wilk Test for normality; *p* > 0.05) and variances of the differences across all groups were equal (Levene’s test for homogeneity for between-subject ANOVA, *p* > 0.05). Statistical analysis was done using the independent values coming from individual animals in each group. One-way ANOVA (between-subjects design) was used to measure the AUC data in open-field locomotion, while two-way repeated measures ANOVA (within-subjects design) were utilized to analyze behavioral and functional effects of different Δ^9^-THC doses on locomotion, analgesia, catalepsy, or body temperature. Post hoc analyses were done using Student-Newman-Keuls method compared to vehicle/baseline control group. The value of *p*< 0.05 was used to indicate statistically significant differences among or between groups. All tests were performed using SigmaStat 12.5 for Windows. The investigators were blinded to the group allocation during the experiments and data analysis.

## Data Availability

All the data are presented in the main manuscript and the additional supporting files. They will be deposited in a publicly available repository (GitHub) after publication.
